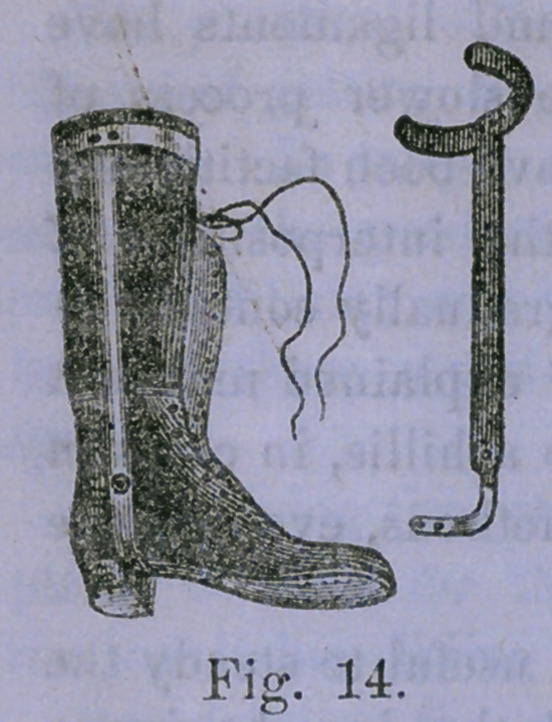# Report on Orthopedic Surgery, Made at the Annual Meeting of Illinois State Medical Society, Convened in Chicago, May 3d, 1864

**Published:** 1865-03

**Authors:** David Prince

**Affiliations:** Jacksonville, Illinois


### REPORT ON ORTHOPEDIC SURGERY,

MADE AT THE ANNUAL MEETING OF THE ILLINOIS STATE MEDICAL SOCIETY, CONVENED IN CHICAGO, MAY 3D, 1864.

By DAVID PRINCE, M. D., Jacksonville, Ill.

(We give in this number of the *Journal* the concluding portion of Dr. Prince's valuable Report, embracing the original and practical parts, with appropriate illustrations. The former portion may be found in the *Journal* for October, November, and December, 1864.—*Editors.*)

### APPARATUS.

In the older plans of treatment, still retained by many of our surgeons of reputation, some immovable and inelastic frames of wood or iron, properly padded, was employed to bring the foot around into proper position; the apparatus being changed for another of different shape as the restoration progressed, or adapted with joints to change with the changing shape of the foot.

The simplest and oldest form is a flat splint, to apply to the leg, with a flat, thin foot-piece, the edge of which was fastened upon the end of the splint, in the form of a cross, upon which the foot and leg was bound by roller-bandages. In contrast with the simplicity of this, are the complicated machines, invented by Scarpa, Scontetten, and others, in the beginning of the great awakening upon the subject of orthopedia, about thirty years ago.

Scarpa's shoe has an iron sole, an iron heel-piece at right angles with this, and a brace running up the leg, while a spring attached to the side of the shoe, gives a pull with some elasticity for straightening the incurved foot; all this is properly padded and provided with straps and buckles. The vertical brace passes up on the projecting or convex side—upon the outer side in talipes varus. The illustration, fig. 5, shows the iron frame-work of the complicated machine.

*Explanation.*

The shoe is in a straight position, *a* the sole, *b* the semi-circular portion to embrace the heel, a portion behind is cut away, leaving a hole for the end of the heel to protrude; *c* the horizontal spring for abduction of the foot; *e* a hinge in the upright portion; *f* a triangular screw-head which is turned with a key, and causes the point of the instrument to turn down; *g* another hinge; *h* another triangular screw-head, which, being turned with a key, bends the foot part outward; *i* the upright shaft or brace; *k* the semi-circular part to go round the leg, and act as a fixed point of the apparatus.

Fig. 5.

Scontetten's apparatus differs from Scarpa's chiefly in having two shafts, one passing up on each side of the leg. Fig. 6 illustrates it without all its padding.

Dr. Bauer, in his work already so often quoted, employs a slight modification of Scontetten's apparatus, as the utmost advance in the art at the present time.

Fig. 6.

These machines, however, are not well adapted to any species but T. equinus and T. varus, and for each varying size of foot, an expensive apparatus must be made. They are uncomfortable, extremely liable to produce ulceration, almost destitute of elasticity, acting chiefly upon the ankle-joint, and moving the foot as a whole, failing to move the tarsal joints upon each other as is done when the foot is grasped by the hand. They are difficult to make except by skilled instrument makers. The desideratum is a method which is within the skill of any person of ordinary ingenuity, to be made of materials always at hand, and free from expensiveness.

The use of adhesive plaster, introduced about the year 1850, was a great advance in the art. The method consists in cutting strips of convenient width and long enough to envelop the foot and pass up the leg nearly to the knee, there to be fastened in place by circular strips passing round the leg, over which the upright strip (or strips, for there usually must be several of them), are turned so as to clinch them to prevent their sliding.

For T. varus the plaster ascends on the outside, and for T. plantaris, and T. valgus on the inside, and for simple T. equinus, on both sides. It is sometimes found convenient to carry the fastening above the knee for greater space for application of the plaster.

This expedient holds the foot in the position in which it is placed by the hand of the surgeon, except a little sliding that plaster will always be guilty of. It very soon occurred to me that a piece of elastic rubber ribbon could be interposed in the vertical strip of adhesive plaster, so as not simply to hold the foot in the position in which it was left by the hand, but to be constantly gaining by a yielding but unintermitting stretch, night and day, gradually elongating the opposing muscles and ligaments, and by the slight mobility attending the elastic rubber, permitting some passive motion in the muscles assisted by the elastic appliance, whereby their circulation is increased, with a more rapid nutrition and a more speedy accommodation to their altered length of contraction.

I for sometime supposed this to be the last advance of which the art was capable, but, ulceration sometimes occurred upon the edge of the foot, where the circulation was too much impeded by the circular compression of the plaster around the foot. There seemed to be a lack of some expedient by which the fold of the tarsus could be straightened out, so as to restore the foot to its normal breadth. An obstinate case, attended with ulceration of a delicate skin, led me to devise an appliance which is a tolerable substitute for the hand ; but before describing it, a few pages must be devoted to the plan of treatment pursued by Mr. Barwell, to explain which, his book (on Club-Foot, &c.) seems to have been chiefly written.

The peculiarity of Borwell's plan consists in his method of attaching the proximal end of his tension apparatus, which is this : Starting with the idea of making the artificial tension the exact complement of that of the partially paralyzed muscles, he aims to act as nearly as possible upon the same bones to which these muscles are attached, (and in the same direction), by adhesive plaster fastenings, while the points from which the pull comes are the origins of these muscles.

Thus, for T. varus, the fastening is made on the exterior anterior side of the upper part of the leg, at a point over the origins of the peronei muscles, in such a way as to get twothirds of the length of the leg for the position of the rubber spring upon which he relies for the pull.

The lower attachment is made to imitate as nearly as possible the insertions of these muscles; but for retention to the skin, the lower adhesive plaster passing downward over the cuboid and fifth metatarsal bones must cross the bottom of the foot, and fasten upon the inner side above the sole. In order to get a retention of the rubber spring upon the upper part of the leg, a broad strip of adhesive plaster, twice the length of the leg, is applied over the course of the peronei muscles, over the fibula, and upon this, a piece of tin, a little narrower than the plaster, is laid, and the lower end of the plaster turned up over it, so that the inside (or sticky side) is outside, for adhering to the roller that applies round the whole to hold it fast. The upper end of the tin is turned over from the leg, and has a hole punched in it, and into this hole an eyelet is inserted; a similar eyelet is inserted in the adhesive plaster which passes across the bottom of the foot, and between these is stretched a rubber spring. By the combination of two or more of these expedients, he is enabled to obtain tension which imitates the combined action of the peroneus longus and p. brevis, passing behind the external malleolus, and the peroneus tertius, passing in front.

For talipes valgus, he makes a similar appliance on the inner side of the leg and foot, to supply the deficiency of the partially paralyzed tibialis anticus and tibialis posticus. The pull must here be in two directions as in the other case.

In talipes plantaris, (flat-foot,) he makes a direct lift upon the hollow of the foot, by an anterior appliance compensating the deficient lift of the tibialis anticus.

In talipes equino dorsaris, he makes also a direct lift further forward. He explains this deformity as being the direct opposite of talipes plantaris, or flat-foot, in which the medio-tarsal joint sinks too low, hence it must be lifted up; while in talipes equino dorsalis, the same joint rises too high, while by the contraction of the tibialis posticus, the peroneus longus, the p. brevis, and the flexor longus digitorum, the metatarsus is flexed or drawn down, bringing the toes to the ground, while again the instep or "waist" of the foot rises too high. He thinks the action of the sural muscles, through the tendo achillis, on the calcaneum, a minor element in the deformity, and hence a particular objection to the division of the tendo achillis, in addition to the general objection arising from permanent injury to the tendon.

The account would be more nearly correct to say, that in addition to the contraction of the tibialis posticus and flexor longus digitorum, the foot is arched too high by the shortened condition of the adductor pollicis, the flexor brevis digitorum perforans, the abductor minimi digiti, and the musculus accessorius, with shortening of the plantor fascia to correspond with this disproportionate contraction of these muscles.

The pull directly in the line of these tendons, besides being a refinement of treatment difficult, and sometimes impossible to execute, is one which acts at a great mechanical disadvantage, implying a greater pressure upon the skin, to accomplish a given amount of change of position, than would be required by a direct pull.

If it had been the design of nature to make only slow movements of the extremities, there would have been nothing gained by binding down the tendons under transverse ligamentous substances as they pass the joints. A much smaller force would have accomplished the purpose, by acting in a straight line between the origin and the insertion of any muscle. The facility of movement and grace of form secured, by giving the tendons oblique attachments, are elements unnecessary to be regarded by the orthopedist. There is this great disadvantage in this attempt to imitate the oblique action of the muscles: that the pressure upon the skin is three or four times what it is necessary to make it, when the most direct pull is obtained. The importance of gaining the most power with the least pressure upon the skin of the foot can hardly be exaggerated. Ulceration of the foot, where the pressure applies, is the greatest difficulty which it has been the study of surgeons to avoid.

It can not be said that the muscle which is partially paralyzed is more assisted by the oblique pull than by the direct, for the passive motion of the muscle is communicated by the push and pull of the tendon; and this *to* and *fro* movement, must be the same for a given amount of motion of the parts to which the tendon is attached, whether the movement is effected by an oblique pull in the direction of the attached end of the tendon, or by a power acting at a less mechanical disadvantage, like the hand of the operator, or any apparatus which acts in a similar manner.

Fig. 7.

Fig. 8.

*Illustrations of Barwell's Method.*

Fig. 7 shows the manner of applying the plaster over the tibia, and the tin over it, and the plaster under the sole of the foot for T. plantaris: *a* a trapezoid piece of plaster into which an eyelet has been fixed, adhering to the sole of the foot, to act as the insertion of the tibialis anticus tendon; *d* a strip of plaster adhering over the tibialis anticus muscle, and having its lower end hanging down more than the length of the limb. The letter *d* is upon the upper portion of this free part; *e* a piece of tin carrying at the top a wire loop; *f* the free end of the plaster is turned up on the tin, and a roller applied to hold all fast.

Figure 8 shows the process completed. The lower end of the long piece of plaster has been turned up over the lower end of the tin, and in the figure circular investments of plaster are shown instead of a roller; *g* strip of plaster surrounding the foot, but leaving out the end of the plaster *b* having an eyelet in it; *l* a rubber spring running from this eyelet in the plaster, which comes from under the sole of the foot, up the leg to the wire loop at the upper end of the tin.

Figure 9 shows the application of the same plan in the treatment of T. varus. Two springs are shown, imitating the action of the peroneus tertius in front of the external malleolus and the peroneus longus, and p. brevis behind the malleolus.

*m* A trapezoid piece of plaster applied across the bottom of the foot and having an eyelet. The course of this under the circular strips is marked by dotted lines *n*. It is represented as being split so as to embrace the fifth metatarsal bone. *n* The eyelet for the attachment of the rubber spring by a piece of catgut or other strong cord. *o* Circular strapping, covering but one piece of tin, placed just behind the fibula, with its layer of plaster on either side. *v* The remainder of the longitudinal strip of plaster brought down and adherent to the circular ones. *t* A rubber spring assisting the peroneus tertius. *u* A rubber spring assisting the p. longus and p. brev. At the lower part of the attachment of the spring, marked *u*, is an arrangement for changing the direction of the force, by an attachment around the limb. *v* A short piece of rubber tube covering a hook, by which the spring is attached to the eyelet in the upper end of the tin. All the attachments are covered in the same way in practice to shield the hooks from the clothes.

Fig. 9.

In obtaining the pull from a space directly over the elongated muscles, by the plaster and tin appliances, a very considerable pressure is produced over the whole circumference of the part. We know that a moderate pressure like that produced in health by the skin and fasciæ, and by a laced stocking, when these are relaxed in varicose veins of the extremities, is favorable to muscular tone, but a greater degree of pressure, like that produced by ligating a member for cramp, is unfavorable to muscular contraction. It is feared that in this method of obtaining the resistance to the pull of the artificial muscle, directly over the muscle whose weakness is to be compensated, there may be a temptation, in hands more unskillful than those of Mr. Barwell, to bind the limb so tightly as to interfere with the most rapid restoration of the muscular function. This tightness is almost necessary, in order to prevent the tin with its underlying adhesive plaster from sliding.

The application of adhesive plaster to the foot, as employed by Barwell, does not materially differ from the method for many years in common use. The plaster cannot be stuck to the skin as the tendon is stuck to the bone. It must have a considerable breadth of attachment or it will slide off. This necessary extent of surface cannot easily be obtained upon the foot without carrying the plaster round upon the opposite edge, so that its pull must approximate the bones of the metatarsus and of the phlanges. This force is the direct opposite of that which is produced upon an inverted club-foot (talipes varus) by walking upon it. The weight of the body, in walking, comes upon the cuboid, the fifth metatarsal bone, and corresponding phalangeal bone until, by folding and twisting, the foot is still further turned, so as to bring the weight of the body upon its dorsum.

The plaster takes hold of the opposite or inner border, (in talipes varus), and passing under the foot and up on the outside pulls in the opposite direction. In all this there is no tendency to take the longitudinal fold or doubling out of the foot. The force simply untwists the malposition of the cuboid in relation to the calcaneum, and the cuneiform bones in relation to the scaphoid, and, more than all the others, the scaphoid in relation to the astragalus. To the extent of the tilting of the astragalus in the ankle-joint, and the sliding of the calcaneum upon the astragalus, these deviations are also corrected.

It is obvious, by a glance at the skeleton, that an important agency in reducing the slight dislocation of the cuniform bones upon the scaphoid, and the principal dislocation of the scaphoid upon the astragalus, is the unfolding of the foot to give it transverse breadth. This is one of the most important indications in cases in which the patients have been some time walking. It is easy enough to answer this indication with the thumb and fingers taking hold of the foot and twisting it in directions opposite to those of the distortion ; but the thumb and fingers soon tire out. It is possible to employ a succession of hands for that purpose, and this would probably be as effectual as any more artificial method. The desideratum is the invention of apparatus which will do what the thumb and fingers can do, and do it without tiring out, and without danger of producing ulceration from the persistency of unyielding pressure. The device to answer this end, without much expense, and in a method so easy of execution that it can be readjusted every day or two, is simply this:

For a patient 10 years old, take a sheet of gutta-percha one-third of an inch thick, or a sufficient number of thinner sheets to make that thickness, long enough to encircle the foot, and wide enough to extend from the middle joint of the phalanges to the medio tarsal articulation, between the scaphoid and astcagalus above, and the cuboid and calcaneum below. Apply upon both surfaces of the gutta-percha an investment of muslin of good strength, and lay the whole, thus preprred, into a pan of water nearly boiling hot. While the softening process is going on, the foot should be wrapped with a roller, protecting the prominent points with pledgets of lint or cotton.

As soon as the gutta-percha is thoroughly softened, it is taken out, still lying between its muslin investments, and so applied that its ends come together on the outside of the foot in talipes varus, where the two extremes of gutta-percha should be welded by pressure between the thumb and fingers, previously dipped into cold water to keep the material from sticking to the fingers.

In talipes valgus the extremes of gutta-percha meet and project on the inner or median side of the foot. While the material is yet warm and yielding, a square piece of pasteboard is laid upon the dorsal surface of the foot with a corresponding piece of oiled silk or rubber cloth, underlying it, to prevent its softening by the moisture of the wet muslin investment, and a similar piece of pasteboard is applied directly opposite upon the plantar surface.

A common pair of calipers, with screw fastening, is then applied, so that one leg rests upon the pasteboard upon the dorsal, and the other upon the pasteboard upon the plantar surface. The screw is then turned to secure very firm squeezing between the opposing points. This compression is continued until the gutta-percha has become hard and unyielding, except by its elasticity. After this the calipers are removed.

A hole is then punched through the projecting gutta-percha, along side of the metatarsal bone of the little toe in varus, and of the great toe in valgus. Into this hole a cord is inserted, which is fastened to a rubber ribbon or piece of rubber tube or cylinder, which must again have its attachment above by adhesive bands below the knee, above tne knee, or by a padded roll to the pelvis which is thereby encircled. This last is the least troublesome attachment, as it can, at any time, be slipped off and put on again. In the last method a knee-cap is necessary to make the tension cord follow the angle of the limb in walking and sitting. The appliance to the foot should be removed and re-applied every day in hot weather, and every alternate day in cold weather, to avoid excoriation from pressure and retained exhalations.

The pressure, if too long applied to a part, without intermission, favors absorption with ulceration; or, if acting with sufficient force, the death of the compressed parts, resulting in sloughing; while the moisture from the skin, with the ammonia which it contains, favors a softening or solution of the cuticle, thus increasing the natural sensitiveness of the parts to pressure.

Figure 10 illustrates the method of applying the apparatus in talipes varus, to secure tension upon the pelvis.

1, Rubber spring. 2, Buckle for adjustment. 3, Gutta-percha investment of the foot, to the outer side of which the tension apparatus is attached. 4, Projection of the toes beyond the investment and above the application of the upper leg of the calipers, applied upon a piece of pasteboard to secure sufficient distribution of pressure. 5, Calipers showing the screw by which the squeezing of the middle portion of the gutta-percha is

Fig. 10.

produced. 6, Knee-bands. 7, Band to which the tension cord is attached, passing obliquely across to the opposite illium. 8, Band around the pelvis to hold the other band from slipping down.

Fig. 11.

Fig. 11 illustrates the same method with an attachment above the knee. It is convenient to have a secondary fastening below the knee which is not shown in the cut.

The figures refer to the same parts as in the preceding cut. The calipers are supposed to have been removed, and the apparatus to have been fully adjusted. The whole may be inclosed in a moccasin or slipper, to enable the patient to walk about. If the patient is an infant, a stocking may be drawn over the apparatus.

Figures 12 and 13 are accurate copies of photographs of a case of talipes

Fig. 12. Before Treatment.

Fig. 13. After Treatment.

varus in a boy nine years old before treatment, and at the conclusion of treatment, at the end of the three months. The flattening down of the tarsus is more perfect than can often be secured without the vertical compression of the foot in the manner just explained. The foot appears shorter than that of the other side, because in the deformed state it had fallen behind the other in growth, but the treatment has spread the foot out effectually, so that there is no danger of a recurrence of the deformity without a nervous derangement capable of producing it from the first.

The following quotation from Barwell, p. 183, aptly illustrates the effect often produced by a theory in hampering one's natural versatility, and driving him to awkward and difficult expedients. The quotation is in explanation of the difficulty of getting room upon an infant's leg for application of plasters, in a child aged six months:

"A little more difficulty" (than usual) "had arisen from the greater adduction of the foot; this rendered it difficult to fasten on so small a thing as a child's leg and foot, the plaster representing the peroneus brevis, so that the end to which the catgut was fixed did not come against the eyelet in the tin representing the pulley. This is a difficulty which always occurs in children's cases. I find it best overcome by cutting the plaster, which is to represent the tendon of a Y shape, stretching it in the hand that it may not give way on the limb, turning down one of the ends, leaving it very short, and fastening the eyelet into it, while the other two ends are made to adhere, one on the sole and one on the dorsum of the foot, leaving the inner metatarsal bone uncovered. In these cases, also, in spite of any difficulty in knotting it, the catgut must be tied very short; the spring, too, must be as short as possible."

In this Barwell recognized, without mentioning or explaining it, the evil of that folding influence upon the foot in talipes varus, arising from pressure of the plaster upon the first metatarsal bone. To avoid this, he stops his dorsal and plantar plasters short of meeting on the tibial side of the foot.

His practical difficulties are very much increased by his theory of getting his pull from over the partially paralyzed muscles. In talipes varus, involving an elongation or loss of action of the peronei muscles, he must get his traction from over the fibula; and he is confined to the length of that bone, because these muscles have only their origins within this space.

By carrying the attachment above the knee there is found plenty of room for the rubber spring, allowing somethg for the inevitable sliding of the plaster.

By adopting the gutta-percha appliance to the foot, a firm fixture is secured equal to a hand continuously applied, which not only does not increase the abnormal transverse doubling of the foot, but helps to flatten it out, thereby much facilitating the rotation of the top or tibial margin of the foot inward or downward, and the bottom or fibular margin outward or upward.

The origin of this theory was in a correct appreciation of the philosophy of the subject, and the failure of the most complete success, grew out of too close an imitation of nature, where power is lost to gain rapidity of movement and beauty of form. In the artificial removal of deformities, rapidity is only the desire of a fool, and beauty is out of the question; while it is of the utmost importance to avoid all unnecessary pressure upon the skin to which the appliances are attached. The more direct the pull, in imitation of the hand of the operator, the lighter will be the pressure upon the skin, the less the discomfort to the patient, and the more practicable the employment of as much force as the muscles and ligaments will bear without pain in these parts.

The fundamental idea which is at the foundation of my plan of treating talipes, is the invention and application of apparatus in imitation of the action of the hand.

Iron shoes and all cumbrous inelastic and expensive machinery are thrown away. The restoration of the proper form of the foot is more likely to be the conclusion of the treatment when the muscles, tendons, and ligaments have been elongated without division, by the slower process of growth from nutrition, than when they have been factitiously elongated by division of tendons, and the interposition of cicatriceal material, material which will gradually contract to complete disappearance. The plan here explained makes it practicable to avoid division of the tendo achillis, in cases in which it might be necessary by the old methods, even by the improved plans of Barwell.

After the treatment is complete, it is useful to steady the foot by a brace running up the side of the leg, having a joint exactly opposite the centre of motion in the ankle. The lower part is made of soft iron, so that the shape can be easily altered, and it is riveted to the sole of a common shoe by two copper rivets, the heads being placed inside the shoe.

The part above the joint, is a flat spring, conveniently made from a worn out saw. The yielding of this spring permits lateral motion at the ankle-joint, while the joint in the apparatus permits flexion and extension. At the top of the spring brace, which should reach about four-fifths of the distance from the ankle to the knee, a cross piece is fastened, made of thick tin or thin iron, of the length of half the circumference of the leg which serves, when bent to the shape of the leg, to prevent the brace from sliding backward and forward. Over the whole length of the elastic portion of the brace, above the ankle, a leather investment of the circumference of the leg and brace is adapted, which is supplied with eyelets to lace upon the opposite side. The brace is always placed upon the side from which the deviation proceeds. The pull is, therefore, from the brace, so that there can never be any chafing of the skin against it. This saves all necessity for cushioning it. The brace is always supporting the ankle-joint, and always yielding as the foot treads upon uneven ground The figures will make this description more intelligible.

Fig. 14.

In figure 14 all portions of the metal above the ankle are invested by the leather, but in the cut, they are represented as being on the outside.

This apparatus will do very well for weak ankles, but should never be trusted after treatment for talipes varus, as long as the instep is in the least too high. The foot should first, not only have the twist entirely taken out of it, but if a T. varus it should not be left in the least degree a T. dorsalis. It is entirely practicable, by the method here described, to convert it into a T. plantaris, but this is neither necessary nor desirable. After this thorough removal of the deformity, the surgeon is not likely to be afterward troubled with the case on account of a tendency to a return of the deviation unless there should be a return of the derangement of innervation, such as originally produced it.

Itmay be noted in closing, that in young infants, previous to walking, and before the infolding of the transverse diameter of the foot from the weight of the body upon its outer margin, the use of the gutta-percha clamp is not very important. The adhesive plaster investment is usually sufficient, but the use of the elastic rubber ribbon is indispensable to satisfactory progress. Where the single ribbon is too delicate, its strength can be increased by doubling. It is convenient to attach a buckle or hook at each end of the rubber ribbon, and to work the adhesive strips into them from above and below. The facility for adjustment is then complete.

In order to obviate the lateral pressure of the plaster upon the foot, a sole of leather may be first applied under the foot, made a little wider than the sole of the foot, and the strips of plaster wrapped around this and the foot combined, as is practised by Dr. H. G. Davis, of New York.

It seems to me that any case of talipes, in a patient under 15 years of age, ought to be restored; but a continuance or a repetition of the derangement of innervation, which originally produced the deformity, may tend to reproduce it, requiring the continued use of an elastic aid to the enfeebled muscles, which may be worn inside of a boot, not differing in principle from the appliances already described, though more delicate and less bulky.

It is not supposed that perfection has yet been attained in this art, nor is it wise to be satisfied with the improvements already made, nor to believe that there is as much known about it now as there ever willbe. If, however, we could see what improvements are to come next, we should immediately make them. Experience feels out the future, but sees the past with eyes open.

Imperfect as may be our present attainments, in this branch of the great art, every child born with uncomplicated talipes, in this and subsequent decades, has that claim for complete restoration at the hands of the profession in his own vicinage, which the accessibility of the knowledge how to do it affords.

A walking specimen of talipes, born after this time, will be a disgrace to somebody, who should have known better.

## Figures and Tables

**Fig. 5. f1:**
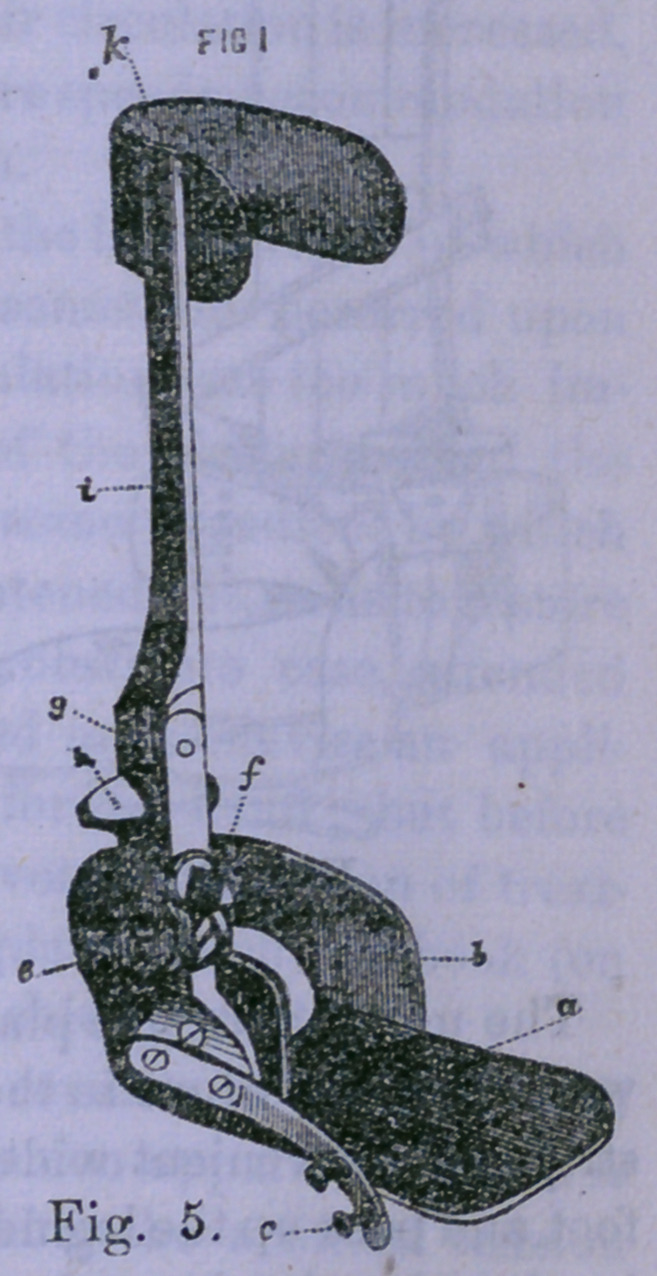


**Fig. 6. f2:**
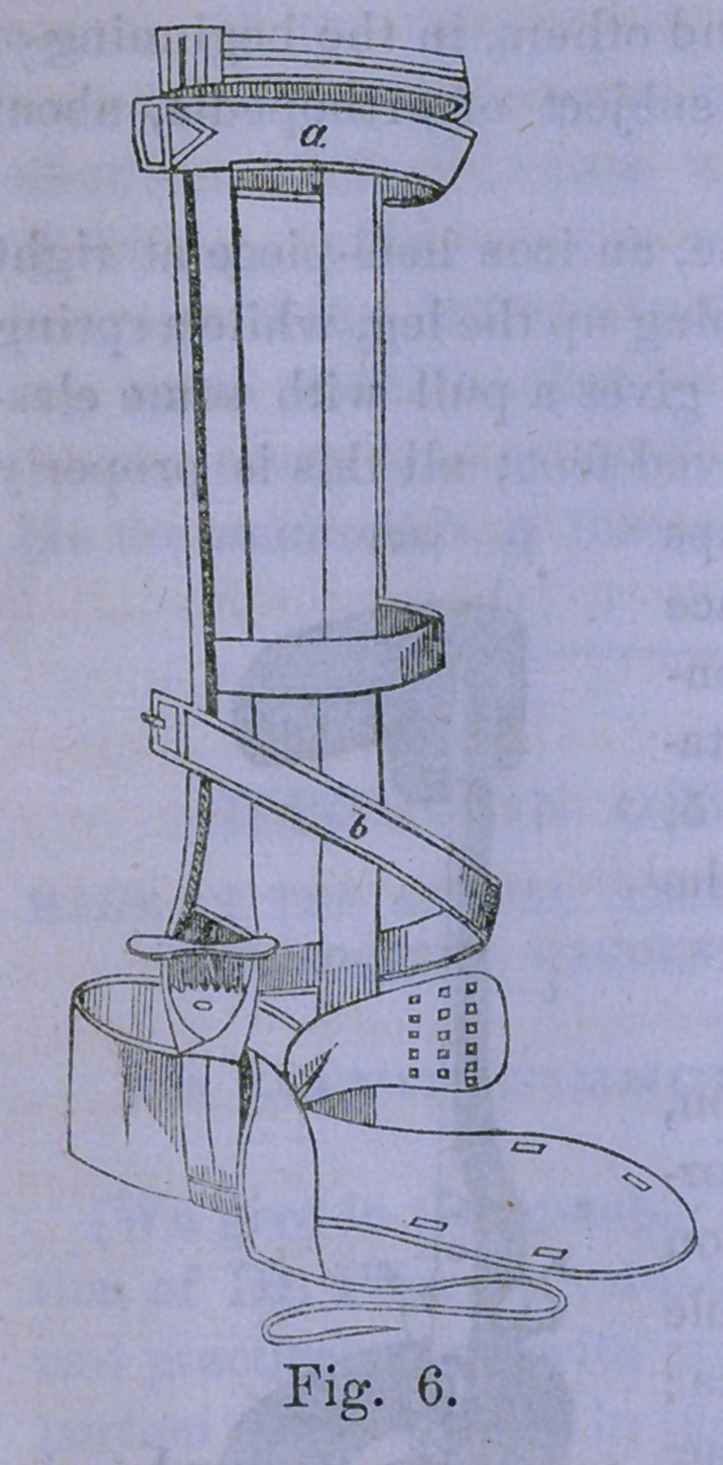


**Fig. 7. Fig. 8. f3:**
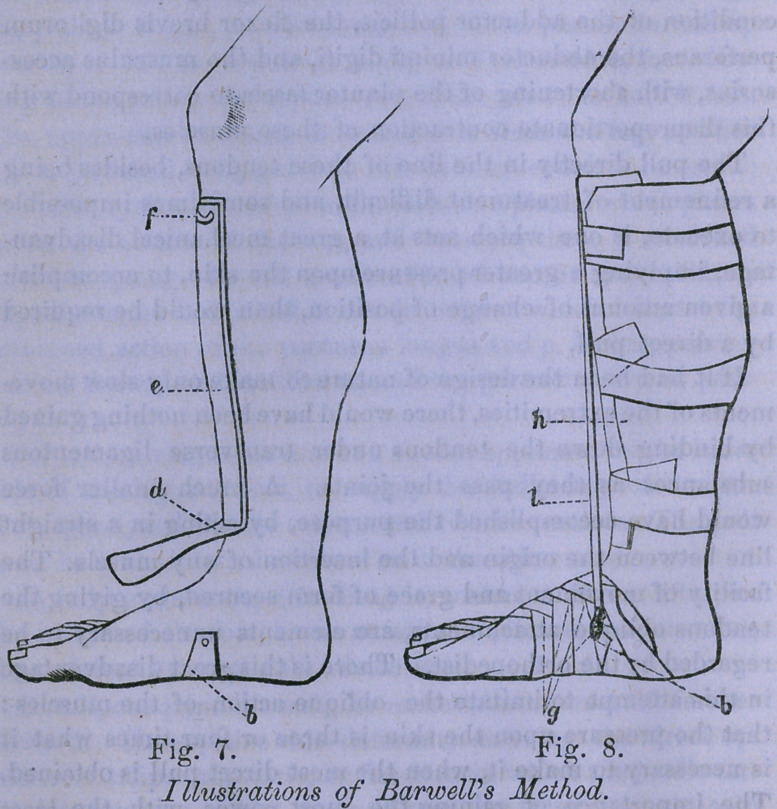


**Fig. 9. f4:**
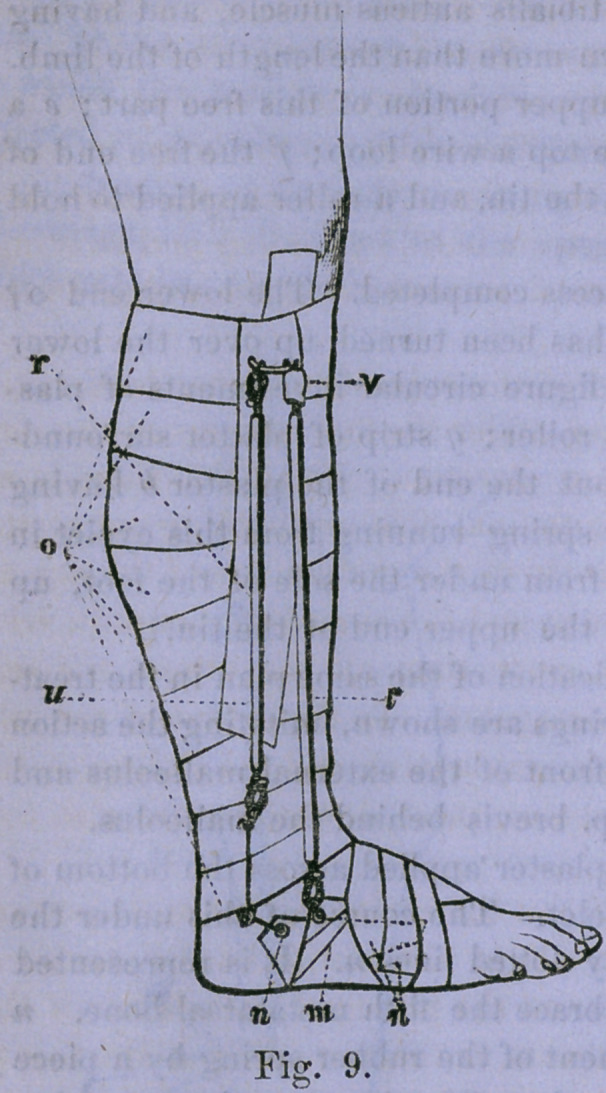


**Fig. 10. f5:**
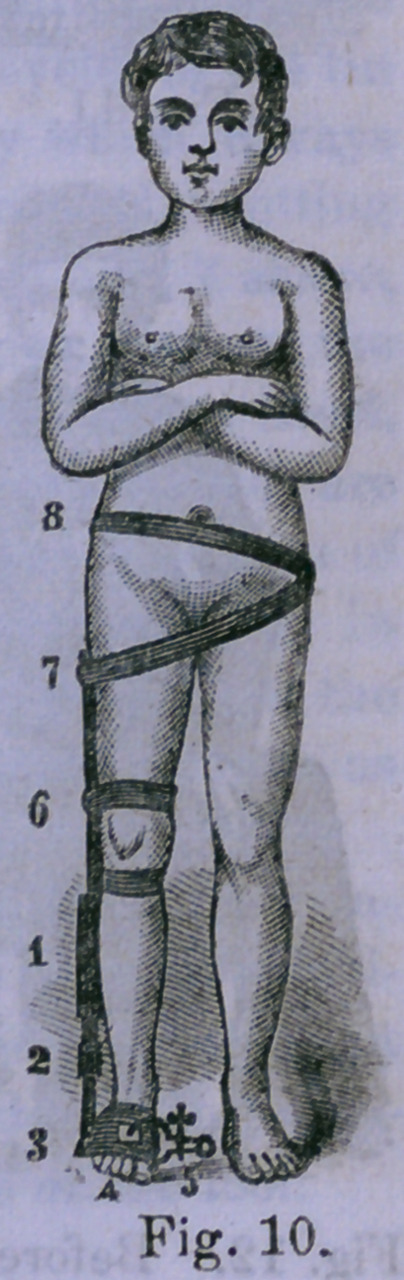


**Fig. 11. f6:**
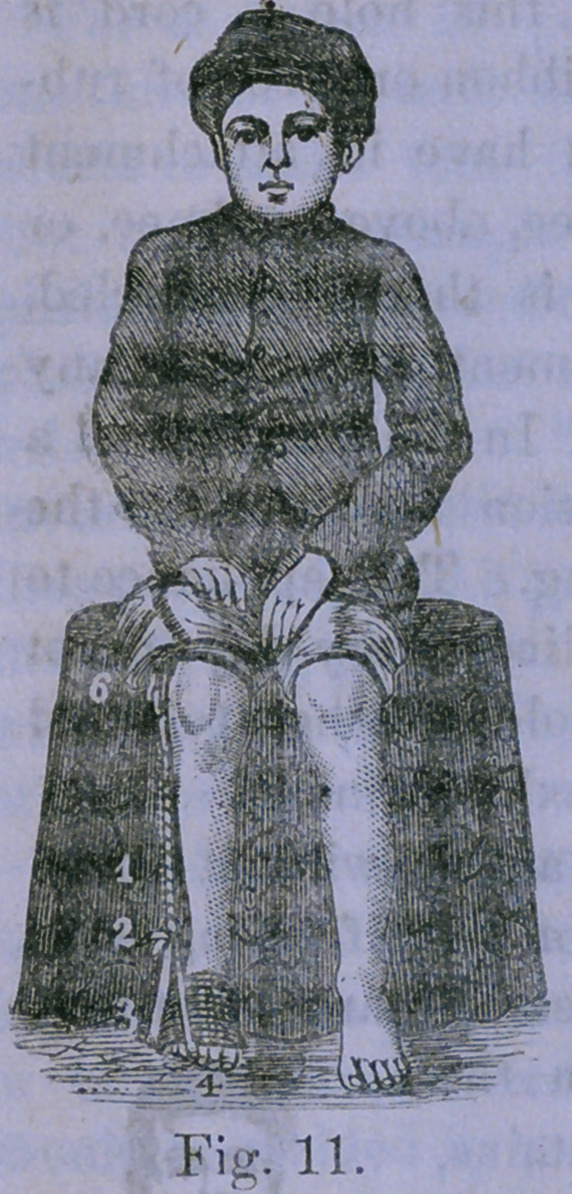


**Fig. 12. f7:**
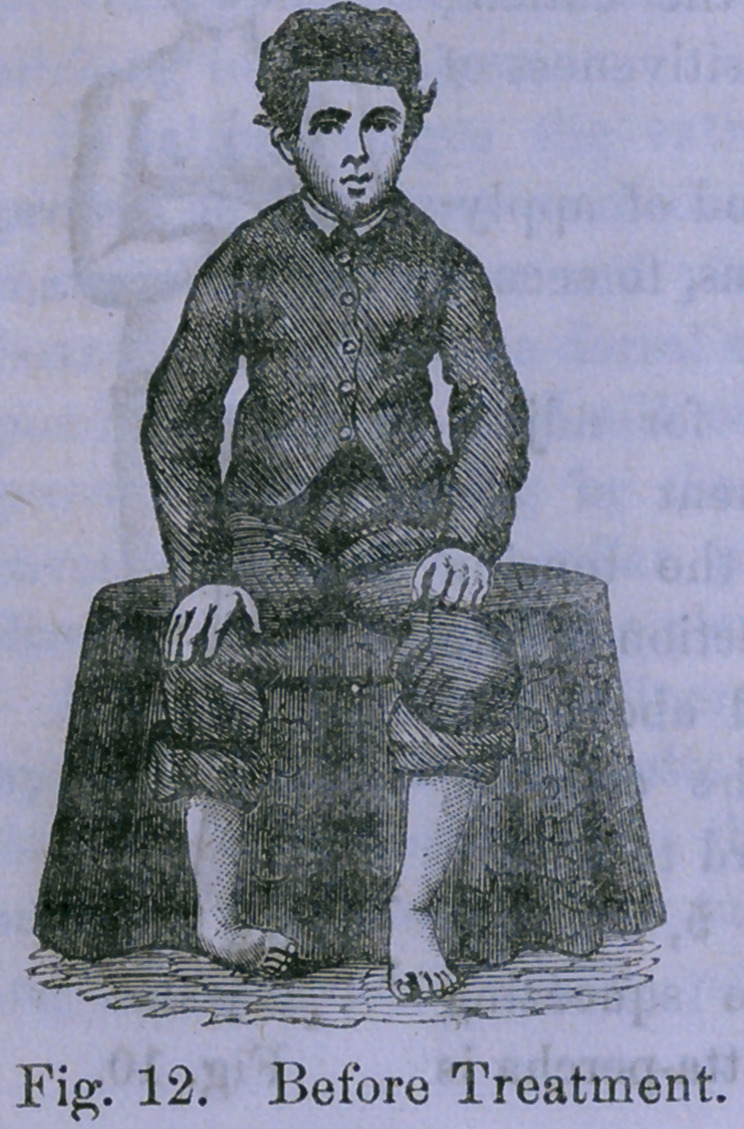


**Fig. 13. f8:**
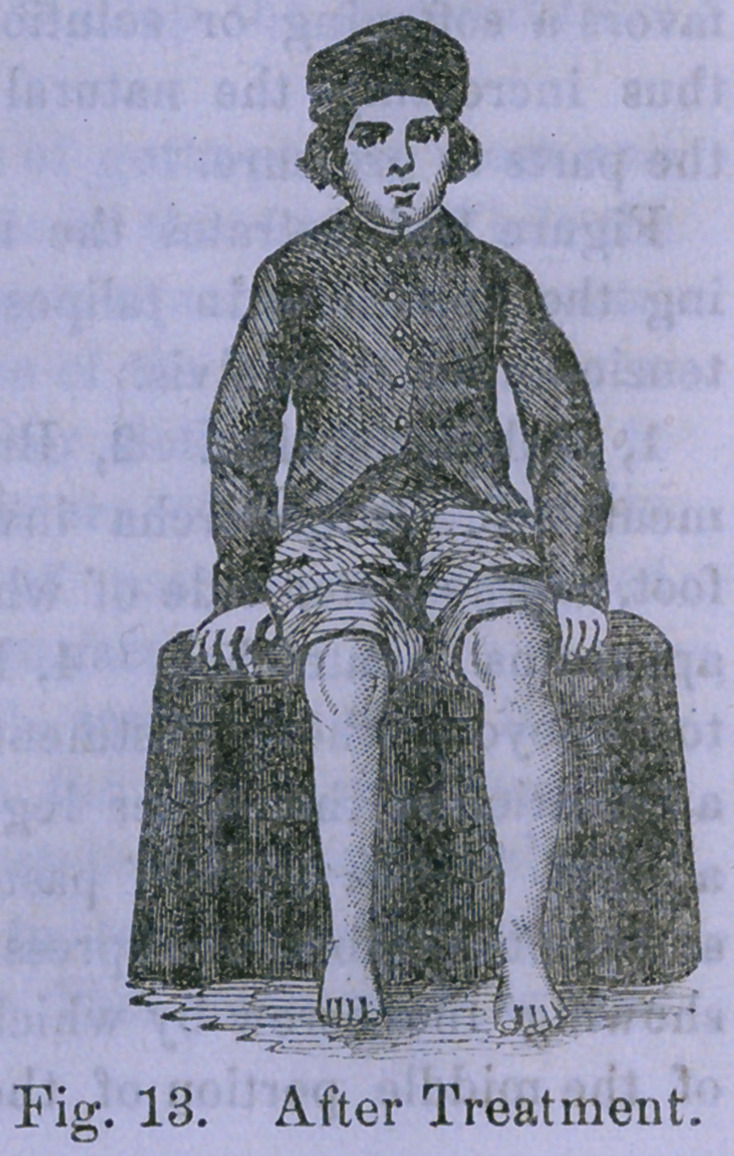


**Fig. 14. f9:**